# Detection of Myosin 1g Overexpression in Pediatric Leukemia by Novel Monoclonal Antibodies

**DOI:** 10.3390/ijms23073912

**Published:** 2022-04-01

**Authors:** Rosa Isela Rodríguez-Téllez, Rosa María Ribas-Aparicio, Genaro Patiño-López

**Affiliations:** 1 Laboratorio de Investigación en Inmunología y Proteómica Hospital Infantil de México, Federico Gómez, Mexico City C.P. 06720, Mexico; rosaalesi_9@yahoo.com.mx; 2 Laboratorio de Producción y Control de Biológicos, Departamento de Microbiología, Escuela Nacional de Ciencias Biológicas, Instituto Politécnico Nacional, Mexico City C.P. 11340, Mexico; rribas233@yahoo.com

**Keywords:** Myo1g, acute lymphoblastic leukemia, monoclonal antibody

## Abstract

Myosin 1g (Myo1g) is a mechanoenzyme associated with actin filaments, expressed exclusively in hematopoietic cells, and involved in various cellular functions, including cell migration, adhesion, and membrane trafficking. Despite the importance of Myo1g in distinct functions, there is currently no monoclonal antibody (mAb) against Myo1g. mAbs are helpful tools for the detection of specific antigens in tumor cells and other tissues. The development of mAbs against targeted dysregulated molecules in cancer cells remains a crucial tool for aiding in the diagnosis and the treatment of patients. Using hybridoma technology, we generated a panel of hybridomas specific for Myo1g. ELISA, immunofluorescence, and Western blot assay results revealed the recognition of Myo1g by these novel monoclonal antibodies in normal and transformed T and B cells. Here, we report the development and application of new monoclonal antibodies against Myo1g for their potential use to detect its overexpression in acute lymphoblastic leukemia (ALL) patients.

## 1. Introduction

Class I myosins are single-headed unconventional myosins whose function involves linking actin filaments to the plasma membrane. This actin–membrane interaction enables them to contribute to diverse functions, such as endocytosis, membrane ruffling, regulation of directional migration, regulation of membrane tension, and other functions, such as vesicular transport [1,2,3,4]. Class I myosins are a subfamily of eight members (Myo1a–Myo1h) characterized by an N-terminal actin-binding ATPase “head” domain, a helical “neck” region that participates in calmodulin binding, and a C-terminal tail region. Class I myosins are divided into “short” and “long” subsets differentiated by the length of their tail region. Both subsets have a tail homology 1 (TH1) region of approximately 200 amino acids evolutionarily conserved [5]. The long tail subset contains an additional Gly/Pro/Ala (GPA) region and an SH3 domain. Myosin I interaction with actin occurs mainly via its N-terminal head domain. The structural basis of myosin I binding to the membrane is less understood. It is mediated by the C-terminal tail region [6]. However, some evidence indicates the participation of the neck region in binding membrane lipids [7]. Later, we describe several regions in the tail that are important for membrane binding, among them a PH domain and regions pre- and post-PH [8]. This is important to understand how Class I myosins achieve their functions.

Leukemia is the most common type of cancer in childhood, particularly affecting children under 15 years old [9], and most prevalent between the ages of 2 to 5 years. Acute lymphoblastic leukemia (ALL) comprises 78% of the cancer cases diagnosed in the pediatric population [10]. This neoplasm is classified into B cell precursors (B-ALL) and T cell precursors (T-ALL), and the immunophenotype of B cell precursors represents nearly 85% of leukemia cases, with the remaining 15% showing a T cell phenotype [11,12]. Risk stratification of patients is a critical component of diagnosis because it helps to decide the appropriate chemotherapeutic treatment. Current treatments have led to a general cure rate of more than 80% in children; however, 15 to 20% of them suffer a relapse. Therefore, finding markers to stratify these patients more efficiently is urgently needed.

Myo1g is a hematopoietic restricted Class I myosin that regulates membrane tension, functions as a “steering wheel” for T lymphocytes, and regulates Fc receptor-mediated phagocytosis in macrophages. It also regulates lipid raft movement, spreading, migration, and antigen presentation in B lymphocytes [4,13,14,15,16,17]. Recently, we found that Myo1g is upregulated in patients with ALL [18]. Myo1g has not previously been directly linked to cancer; although, other Class I myosins have gained interest in the field because some function as tumor suppressors, while others are overexpressed in different cancers [19,20,21]. Furthermore, to date, there are no commercial monoclonal antibodies to Myo1g. As such, our goal was to generate monoclonal antibodies directed against human Myo1g, as tools to better understand the function of Myo1g in lymphocyte biology.

## 2. Results

### 2.1. Production of Anti-Human Myo1g Monoclonal Antibodies

**Figure 1:**
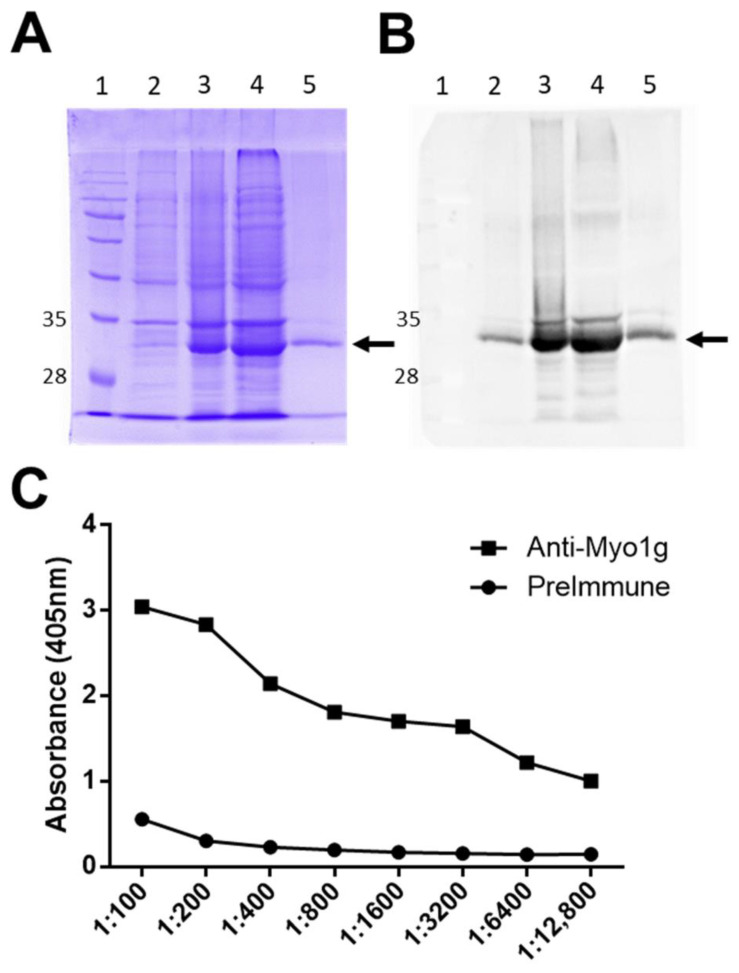
Production and purification of recombinant human Myo1g IQ-Tail and antibody induction in mice. (**A**) SDS-PAGE analysis of rhMyo1g IQ-Tail protein in lysates from control culture (2), induced culture (3), insoluble fraction (Pellet) (4), and purified protein (5) in a 12% gel stained with Coomassie brilliant blue R-250, MW Markers (1). (**B**) WB analysis of His-tagged proteins in an identical distribution as (**A**), the membrane was analyzed by chemiluminescence. Molecular weight of Myo1g IQ-Tail = 34 kDa is indicated by an arrow (**A**,**B**). (**C**) Antibody titers of one mouse after five immunizations with the rhMyo1g IQ-Tail protein.

To generate anti-human Myo1g monoclonal antibodies, a His-tagged recombinant protein encompassing the neck and the tail and produced by *E. coli* via IPTG induction was produced. The recombinant protein was purified from bacterial lysates using Cobalt-IDA-Agarose, and examined by SDS-PAGE (Figure 1A) and WB using an anti-His antibody (Figure 1B). Subsequently, mice with the protein emulsified in Freund’s adjuvant were immunized. After three and five boosting immunizations, mice sera were evaluated by ELISA using the recombinant protein. The results show a significant induction of IgG anti-recombinant human-Myo1g antibody activity (Figure 1C).

**Figure 2:**
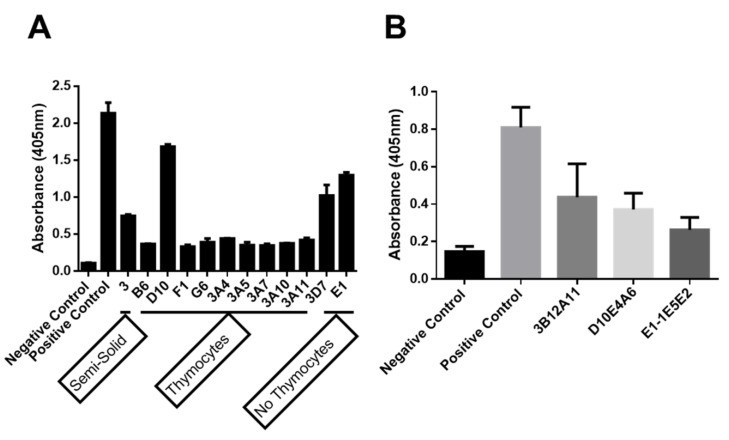
Development of mAbs against Myo1g. (**A**) ELISA results from the original plating after fusion. The addition of thymocytes as feeder cells increased the frequency of positive cells after screening. There were nine positive wells with thymocytes, two without thymocytes, and one in semi-solid media. (**B**) After two rounds of single cell cloning, three individual hybridomas producing high amounts of antibody against Myo1g, positive control (1:6400) and hybridomas (1:50), were established. Representative data from two independent experiments are shown as mean + SD of triplicate samples.

From the mice with the highest titers, a final booster in PBS was administered, and after three days, spleen cells to fuse with Sp2ab myeloma cells were obtained. The cells were divided between three treatments and plated in two 96-well plates, with and without feeder thymocytes (1 × 10^5^/well). A third group was plated in semisolid media. 12 ELISA positive supernatants for rhMyo1g IQ-Tail were obtained. Interestingly, nine positive wells from cells with thymocytes were obtained, two without thymocytes, and one from the semi-solid media (Figure 2A). From those SN, three hybridomas were selected for single cell cloning, and after two rounds, three clones were used for subsequent characterization (3B12A11, D10E4A6, E1-1E5E2) (Figure 2B). Isotype determination from the three mAbs showed that all were IgG1-kappa positive (IgG1-κ).

#### 2.1.1. The New Monoclonal Antibodies Detect Endogenous Myo1g

**Figure 3:**
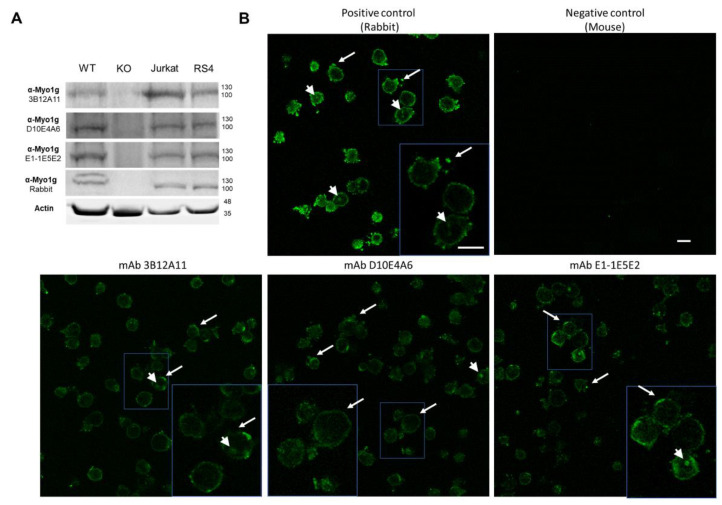
Endogenous Myo1g detection with the newly developed monoclonal antibodies. (**A**) Western blot analysis of spleen cell lysates from WT and Myo1g KO mice, and lysates from Jurkat (human T-ALL cells) and RS4:11 (human B-ALL cells) using the three monoclonal antibodies (3B12A11, D10E4A6, E1-1E5E2), and a purified rabbit IgG anti-Myo1g and B-actin as loading control. (**B**) Top left panel, representative confocal images of Jurkat T cells stained with the purified rabbit IgG, specific to Myo1g, positive control; top right panel, negative control without primary antibody; lower panels, supernatant from the Myo1g-specific monoclonal antibodies. Arrows indicate Myo1g expression at the plasma membrane or membrane extensions; arrowheads indicate Myo1g expression inside the cells; scale bars = 10 µm.

Myo1g is abundantly expressed in T and B lymphocytes [8,22]. To demonstrate whether the anti-Myo1g mAb recognized endogenous Myo1g in human and mouse cells, we performed WB analysis with lysates of spleen cells from C57BL/6 WT and Myo1g KO mice, and from Jurkat (human T-ALL) and RS4-11 (human B-ALL) cell lines. The results showed that the anti-Myo1g mAb recognized Myo1g in the cell lysates, from the WT mouse, and the human cell lines, but not in those from the KO mouse (Figure 3A). A purified rabbit IgG directed against the N-terminal of Myo1g [8] was used. Jurkat cells were stained and evaluated for the expression of Myo1g. It has been reported that Myo1g is mainly located at the plasma membrane, although there is some expression in vesicles. The staining with our three monoclonal antibodies compared with the positive control revealed the expected expression occurred mainly at the plasma membrane (Figure 3B). Interestingly, the mAb 3B12A11 exhibited a clearer staining at the membrane. This antibody was further evaluated using ALL samples from pediatric patients to demonstrate the potential of Myo1g as a biomarker.

#### 2.1.2. Myo1g Overexpression in Pediatric Patients with Acute Lymphoblastic Leukemia

**Figure 4:**
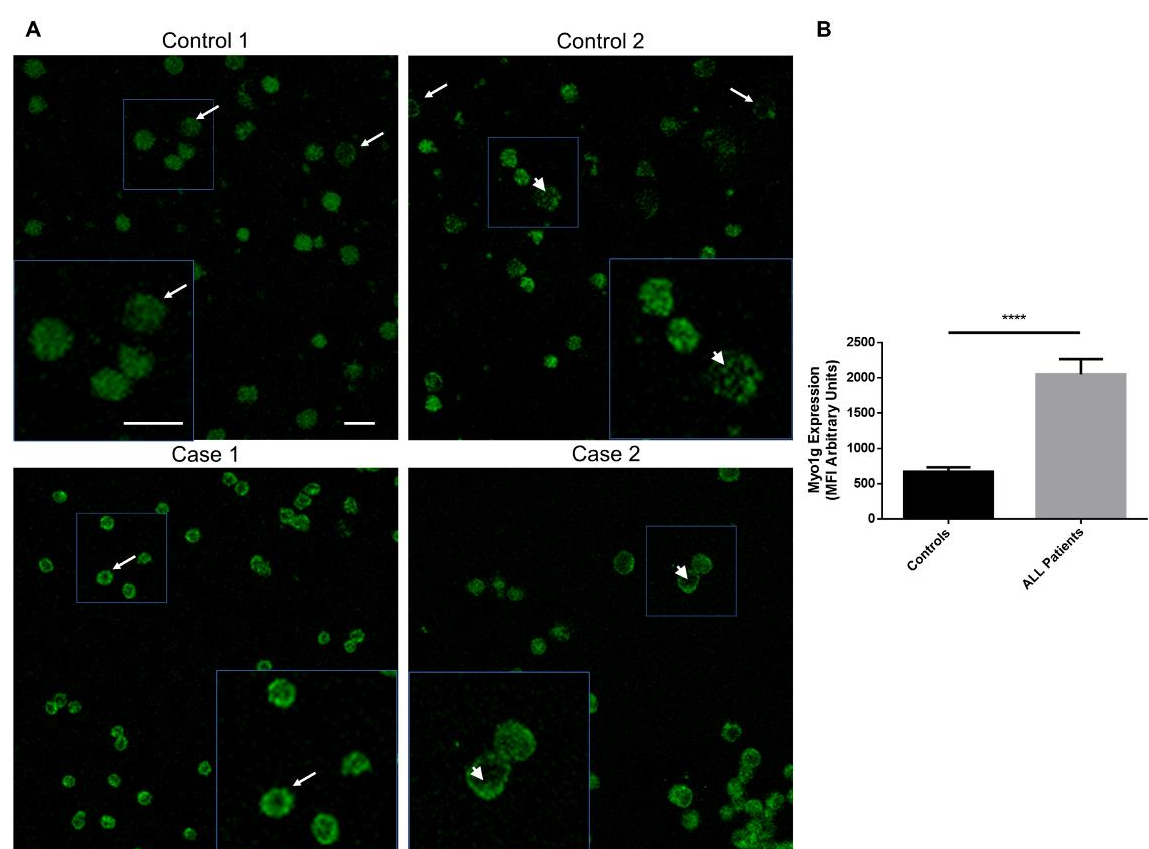
Myo1g detection in pediatric acute lymphoblastic leukemia patients. (**A**) Representative confocal images of the expression of Myo1g in two controls and two ALL patients. Arrows indicate Myo1g expression at the plasma membrane or membrane extensions; arrowheads indicate Myo1g expression inside the cells; scale bars = 10 µm. (**B**) Mean fluorescence intensity (MFI) of Myo1g expression in PBMCs from three controls and seven patients (*n* = 71 control cells and *n* = 159 patient cells). **** *p* < 0.0001; two-tailed unpaired Student’s *t*-test; graph bars = 95% CI.

Myo1g is exclusively expressed in hematopoietic cells [8,22]. Recently, we showed that Myo1g is overexpressed in pediatric ALL patients, and proposed that Myo1g is a potential high-risk biomarker. Based on these results, we decided to evaluate whether the recently generated monoclonal antibodies detect the endogenous Myo1g expression in ALL patients. To test this hypothesis, we used PBMCs from seven patients, and compared their expression with three pediatric control PBMCs through fluorescence intensity. In Figure 4A, representative confocal images of Myo1g expression in two patients and two controls are shown. By quantifying the expression in multiple cells from each group, we found that the higher Myo1g expression is statistically significant in cells from patients compared with that in control cells (Figure 4B). These results show the potential use of the newly generated monoclonal antibodies in the detection of Myo1g as a biomarker in pediatric ALL.

## 3. Discussion

Myosin 1g is highly expressed in B and T lymphocytes; recently we demonstrated its overexpression in high-risk pediatric ALL patients [8,18,23]. In this study, we generated monoclonal antibodies against human Myo1g, and demonstrated that the three of them could recognize the endogenous protein in normal and transformed B and T cells, and evaluated their potential use in recognizing the overexpression of Myo1g in blood samples from pediatric ALL patients as a putative biomarker.

The Myosin family has a highly conserved motor domain, and the main differences among its members are located in the tail domain [24,25]. We produced a recombinant protein comprising the tail and IQ domains of Myo1g, and successfully induced specific antibodies against Myo1g, evident by its lack of detection in cells harvested from Myo1g-deficient mice. Many of the Class I myosins were expressed in mice spleen cells, including Myo1b, c, e, f, and g [8,23]. Interestingly confocal microscopy staining revealed a similar pattern of expression of Myo1g in primary cells and transformed cell lines.

Myo1g participates in different cell functions, particularly regulating membrane tension and Fc receptor-mediated phagocytosis, controlling cell migration and the turning angles of T and B cells, especially under antigen-limited conditions [13,14,16,26]. Additionally, we demonstrated that Myo1g could be used as a high-risk biomarker in pediatric ALL [18]. Leukemia treatment has improved in recent years; however, 15–20% of patients relapse [27,28]. Therefore, identifying new biomarkers that could help to stratify the patients and improve their treatment outcomes and quality of life is of utmost importance. The gold standard for diagnosis of ALL involves the analysis of bone marrow aspirates to determine the presence of blasts, is complemented with cytogenetics, and molecular and immunological studies; however, the invasiveness of the procedure still limits the clinical application of the current methods [29]. Therefore, it is necessary to find new biomarkers that are noninvasive and specialized for the early diagnosis of the disease. At present, we are designing a study to evaluate the possible use of Myo1g in blood samples of patients to determine the usefulness as a diagnostic marker; in addition, we plan to design an ELISA test to determine the expression of Myo1g in serum or plasma samples from patients to determine its diagnostic use in ALL.

Class I myosins are a family of monomeric unconventional myosins, comprised of eight members (Myo1a–h). There are recent reports of its deregulation in different cancer types; for example, Myo1a seems to be a tumor suppressor in the intestine [30], Myo1e promotes tumor proliferation and cell de-differentiation in breast cancer [31], and Myo1b overexpression promotes increased cell migration and lymph node metastasis in head and neck cancer [32]. Despite its importance, only Myo1e has monoclonal antibodies available in the market for cancer treatment, as revealed by a thorough search among the main manufacturers. Beyond our report of Myo1g overexpression in ALL, there are no other reports of the functional implications of Myo1g in cancer; however, such tools as our newly generated antibodies, here reported, could shed light on currently unknown functions of this family of unconventional myosins. The main goal of this study was to generate monoclonal antibodies against human Myo1g; this protein is being actively studied by different groups, including ours, and has been demonstrated to be important for various immune cell functions. Furthermore, we recently demonstrated that Myo1g has the potential for use as a high-risk marker in pediatric ALL.Here, we have demonstrated the potential utility of these monoclonal antibodies in patient samples. We will continue to characterize and provide further evidence for their potential use, for example, in ELISA assays, among other tests.

## 4. Materials and Methods

### 4.1. Purification of Recombinant Human Myo1g IQ-Tail Protein

The human Myo1g IQ-Tail was described previously [8]. Briefly, the construct comprising aa 702–1018 was subcloned into pDes17 by Gateway cloning (Invitrogen, Carlsbad, CA, USA), which generates 6x His-tagged N-terminal proteins. The plasmids were transformed into *Escherichia coli BL21DE3* (Thermo Scientific, Waltham, MA, USA) competent cells, and protein expression was induced with 1 mM isopropyl β-D-1-thiogalactopyranoside (IPTG) (Promega, Madison, WI, USA) for 5 h at 37 °C. Cells were lysed by sonication in lysis buffer (300 mM NaCl, 50 mM NaH_2_PO_4_, 10 mM imidazole, 6 M urea, pH 8, and a protease inhibitor cocktail-EDTA free (Thermo Scientific, Rockford, IL, USA) on ice. After centrifugation, cell lysates were purified by the batch method using 6% Cobalt-IDA Agarose (Jena Bioscience, Jena, Germany) under constant agitation at 4 °C overnight, followed by 4 washes (300 mM NaCl, 50 mM NaH_2_PO_4_, 10 mM imidazole, 6 M urea, pH 8). Bound proteins were eluted with elution buffer (300 mM NaCl, 50 mM NaH_2_PO_4_, 350 mM imidazole, 6 M urea, pH 8), and the filtrate was concentrated with Amicon Ultra-15 (Merck Millipore, Cork, Ireland) through a 0.22 µm mesh, and analyzed by SDS-PAGE and Western blot.

### 4.2. Cell Lines

Sp2ab cells (Enzo, Farmingdale, NY, USA) were cultured in PCM1x medium (Enzo, Farmingdale, NY, USA). Jurkat TAg (donated by Dr. Stephen Shaw, NCI, NIH, Bethesda, MD, USA) and RS4:11 cells were cultured in RPMI-1640 (BioWest, MO, USA) supplemented with 10% FBS (BioWest, Riverside, MO, USA), 100 U/mL penicillin, and 100 mg/mL streptomycin. All cell lines were maintained at 37 °C in 5% CO_2_. Myo1g-deficient mice have been described elsewhere [13]; here we used spleen lysates from age- and sex-matched C57BL6 mice as controls, kindly donated by Dr. Leopoldo Santos-Argumedo (Molecular Biomedicine Department, CINVESTAV)

### 4.3. Mice Immunization

Female BALB/c mice (5–6 weeks old) were obtained and maintained in the animal facility of the Hospital Infantil de Mexico Federico Gomez. The animal handling and experimental procedures were approved by the Ethics, Biosafety and Research Committee of the Hospital Infantil de Mexico (Protocol HIM 2015-005 SSA 1182). Mice were intraperitoneally immunized at two-week intervals using 100 µg of the recombinant protein in complete Freund’s adjuvant (Sigma-Aldrich, St Louis, MO, USA), followed by 5 immunizations with 100 µg of the Myo1g IQ-Tail protein in incomplete Freund’s adjuvant (Sigma-Aldrich, St Louis, MO, USA). Four days after the last immunization, spleen cells were harvested and fused with Sp2ab cells at a 5:1 proportion with 50% PEG (Enzo, Farmingdale, NY, USA). Fused cells were seeded in Abeoclone semi-solid media (Enzo, Farmingdale, NY, USA) or plated in 96-well culture plates in the presence or absence of thymocytes (100 thousand cells per well) in RPMI media supplemented with 20% FBS, 1X HAT supplement, 25 mM HEPES, L-glutamine, sodium pyruvate (all from Gibco, Grand Island, NY, USA), and 1x antibiotic–antimycotic solution (Corning, Corning, NY, USA). After approximately 2 weeks, the resulting hybridomas were subcultured in medium supplemented with HT (Gibco, Grand Island, NY, USA) and tested by ELISA for recognition of the Myo1g IQ-Tail. The hybridomas that recognized the Myo1g IQ-Tail recombinant protein were cloned by 2 rounds of limiting dilution, and preserved in liquid nitrogen. The antibody isotype was determined using the mouse monoclonal isotyping kit (Cat. 11493027001, Roche, Mannheim, Germany).

### 4.4. ELISA

Recombinant human Myo1g IQ-Tail (0.02 µg/µL) was immobilized in 96-well immuno-plates (Costar, Kennebunk, ME, USA), and blocked with 5% low-fat milk in PBS with 0.05% Tween 20 (Sigma-Aldrich, St Louis, MO, USA) (PBS-T). Mouse sera and hybridoma supernatant were diluted 2-fold in PBS-T and incubated at room temperature (RT) for 1 h. Afterwards, the plates were washed 3 times with PBS-T and incubated at RT for 1 h with HRP-conjugated goat anti-mouse IgG (H+L) (Cat. ab97023, Abcam, Cambridge, UK), washed three times with PBS-T, after which ABTS substrate was added (Roche, Mannheim, Germany) for 25 min. The reaction was stopped with 1% SDS, and the optical density was measured at 405 nm using a Multiskan FC spectrophotometer (Thermo Fisher Scientific, Shanghai, China).

### 4.5. Western Blot

For Western blot (WB) analysis, equal amounts of protein (50 µg) in RIPA buffer from each cell type were resolved by 12% SDS-PAGE under reducing conditions. Gels were transferred to nitrocellulose membranes (Bio Rad, Hercules, CA, USA), blocked with 5% non-fat milk, washed with PBS 0.05% Tween 20 (*v*/*v*) (PBS-T), and then incubated for 1 h at RT with a 1:1000 dilution of purified rabbit IgG to Myo1g as a positive control and a 1:4000 dilution of supernatant (SN) from the 3 mAb to Myo1g in PBS-T, or a 1:5000 dilution for anti-actin mAb (Sigma-Aldrich, St Louis, MO, USA). Subsequently, they were washed 3 times with PBS-T, and incubated for 1 h at RT with a 1:15,000 dilution of HRP-coupled secondary Ab GAR (Abcam, Cambridge, UK), 1:20,000 of GAM (Abcam, Cambridge, UK). After 3 further washes with PBS-T, luminol reagents were added (Santa Cruz Biotechnology, Santa Cruz, CA, USA). The chemiluminescence analysis was performed on a Fusion FX-7 Spectral Imaging System (Vilber, Paris, France).

### 4.6. Immunofluorescence Analysis

PBMCs from control individuals and ALL patients were adhered to glass slides as reported in [18]. Briefly, 50 µL spots with 10,000 PBMCs were left to dry, the cells were fixed with 50 µL of 4% paraformaldehyde (PFA) (*v*/*v*) and stored until use. 5 × 10^3^ Jurkat T cells and 5 × 10^3^ RS4:11 B cells, both in 50 µL, were left to adhere for 50 min; then fixed with 4% PFA, permeabilized with 0.5% Triton X-100 (Sigma-Aldrich, St Louis, MO, USA) in PBS, blocked with 1% BSA in PBS (PBA), and stained with purified rabbit IgG to Myo1g (1:500 in PBA) or culture SN (1:300 in PBA) corresponding to mAb 3B12A11, D10E4A6 and E1-1E5E2 ON at 4 °C in a humid chamber. After washing three times for 5 min each with PBS-T, secondary Ab was added to either Rabbit IgG or Mouse IgG, conjugated with Alexa 488 (Jackson Immuno Research, West Groove, PA, USA) at a1:500 dilution in PBA for 1 h at RT protected from the light. Subsequently, they were washed 3 times for 5 min each with PBS-T, the nuclei were stained with DAPI in anti-fade, Vectashield mounting medium (Vector Laboratories, Burlingame, CA, USA). The cells were imaged on a Leica TCS/SP8x confocal microscope using a 63x objective and acquiring Z-stacks covering the whole cells with 0.5 µm steps. Fluorescence intensity was quantified from 50 to 100 cells per patient at diagnosis. Images were analyzed using the Fiji package of ImageJ software (NIH); specifically, a line was drawn in the periphery of each cell to measure the mean fluorescence intensity (MFI). Results are expressed as the MFI per cell using arbitrary units for quantification.

### 4.7. Statistical Analysis

Myo1g expression in patient’s cells was analyzed using a two-tailed unpaired Student’s *t*-test. *p*-values < 0.05 were considered significant. Values were expressed as means + SD unless otherwise specified.

## Data Availability

The data presented in this study are available on request from the corresponding author.

## References

[1] Cheney R.E., Mooseker M.S. (1992). Unconventional myosins. Curr. Opin. Cell Biol..

[2] Olivert T.N., Berg J.S., Cheney R.E. (1999). Tails of unconventional myosins. Cell. Mol. Life Sci..

[3] Kim S.V., Flavell R.A. (2008). Myosin I: From yeast to human. Cell. Mol. Life Sci..

[4] Nambiar R., McConnell R.E., Tyska M.J. (2009). Control of cell membrane tension by myosin-I. Proc. Natl. Acad. Sci. USA.

[5] Hammer J.A. (1991). Novel myosins. Trends Cell Biol..

[6] Adams R.J., Pollard T.D. (1986). Propulsion of organelles isolated from Acanthamoeba along actin filaments by myosin-I. Nature.

[7] Hokanson D.E., Ostap E.M. (2006). Myo1c binds tightly and specifically to phosphatidylinositol 4,5-bisphosphate and inositol 1,4,5-trisphosphate. Proc. Natl. Acad. Sci. USA.

[8] Patino-Lopez G., Aravind L., Dong X., Kruhlak M.J., Ostap E.M., Shaw S. (2010). Myosin 1G is an abundant class I myosin in lymphocytes whose localization at the plasma membrane depends on its ancient divergent pleckstrin homology (PH) domain (Myo1PH). J. Biol. Chem..

[9] Pérez-Saldivar M.L., Fajardo-Gutiérrez A., Bernáldez-Ríos R., Martínez-Avalos A., Medina-Sanson A., Espinosa-Hernández L., Flores-Chapa J.D.D., Amador-Sánchez R., Peñaloza-González J.G., Álvarez-Rodríguez F.J. (2011). Childhood acute leukemias are frequent in Mexico City: Descriptive epidemiology. BMC Cancer.

[10] Miller K.D., Nogueira L., Mariotto A.B., Rowland J.H., Yabroff K.R., Alfano C.M., Jemal A., Kramer J.L., Siegel R.L. (2019). Cancer treatment and survivorship statistics, CA. Cancer J. Clin..

[11] Siegel R.L., Miller K.D., Jemal A. (2019). Cancer statistics, CA. Cancer J. Clin..

[12] Ward E., DeSantis C., Robbins A., Kohler B., Jemal A. (2014). Childhood and adolescent cancer statistics, CA. Cancer J. Clin..

[13] Gerard A., Patino-Lopez G., Beemiller P., Nambiar R., Ben-Aissa K., Liu Y., Totah F.J., Tyska M.J., Shaw S., Krummel M.F. (2014). Detection of rare antigen-presenting cells through T cell-intrinsic meandering motility, mediated by Myo1g. Cell.

[14] Dart A.E., Tollis S., Bright M.D., Frankel G., Endres R.G. (2012). The motor protein myosin 1G functions in FcγR-mediated phagocytosis. J. Cell Sci..

[15] López-Ortega O., Santos-Argumedo L. (2017). Myosin 1g Contributes to CD44 Adhesion Protein and Lipid Rafts Recycling and Controls CD44 Capping and Cell Migration in B Lymphocytes. Front. Immunol..

[16] Maravillas-Montero J.L., López-Ortega O., Patiño-López G., Santos-Argumedo L. (2014). Myosin 1g regulates cytoskeleton plasticity, cell migration, exocytosis, and endocytosis in B lymphocytes. Eur. J. Immunol..

[17] Cruz-Zárate D., López-Ortega O., Girón-Pérez D.A., Gonzalez-Suarez A.M., García-Cordero J.L., Schnoor M., Santos-Argumedo L. (2021). Myo1g is required for efficient adhesion and migration of activated B lymphocytes to inguinal lymph nodes. Sci. Rep..

[18] Estrada-Abreo L.A., Rodríguez-Cruz L., Garfias-Gómez Y., Araujo-Cardenas J.E., Antonio-Andrés G., Salgado-Aguayo A.R., Orozco-Ruiz D., Torres-Nava J.R., Díaz-Valencia J.D., Huerta-Yépez S. (2021). High expression of Myosin 1g in pediatric acute lymphoblastic leukemia. Oncotarget.

[19] Ouderkirk J.L., Krendel M. (2014). Non-muscle myosins in tumor progression, cancer cell invasion, and metastasis. Cytoskeleton.

[20] Peckham M. (2016). How myosin organization of the actin cytoskeleton contributes to the cancer phenotype. Biochem. Soc. Trans..

[21] Li Y.-R., Yang W.-X. (2016). Myosins as fundamental components during tumorigenesis: Diverse and indispensable. Oncotarget.

[22] Olety B., Wälte M., Honnert U., Schillers H., Bähler M. (2010). Myosin 1G (Myo1G) is a haematopoietic specific myosin that localises to the plasma membrane and regulates cell elasticity. FEBS Lett..

[23] Santos-Argumedo L., Maravillas-Montero J.L., López-Ortega O. (2013). Class I myosins in B-cell physiology: Functions in spreading, immune synapses, motility, and vesicular traffic. Immunol. Rev..

[24] Krendel M. (2005). Myosins: Tails (and Heads) of Functional Diversity. Physiology.

[25] O’Connell C.B., Tyska M.J., Mooseker M.S. (2007). Myosin at work: Motor adaptations for a variety of cellular functions. Biochim. Biophys. Acta-Mol. Cell Res..

[26] López-Ortega O., Ovalle-García E., Ortega-Blake I., Antillón A., Chávez-Munguía B., Patiño-López G., Fragoso-Soriano R., Santos-Argumedo L. (2016). Myo1g is an active player in maintaining cell stiffness in B-lymphocytes. Cytoskeleton.

[27] Portell C.A., Advani A.S. (2014). Novel targeted therapies in acute lymphoblastic leukemia. Leuk. Lymphoma.

[28] Meyer L.H., Eckhoff S.M., Queudeville M., Kraus J.M., Giordan M., Stursberg J., Zangrando A., Vendramini E., Möricke A., Zimmermann M. (2011). Early Relapse in ALL Is Identified by Time to Leukemia in NOD/SCID Mice and Is Characterized by a Gene Signature Involving Survival Pathways. Cancer Cell.

[29] Shahid S., Shahid W., Shaheen J., Akhtar M.W. (2021). Circulating miR---146a expression as a non---invasive predictive biomarker for acute lymphoblastic leukemia. Sci. Rep..

[30] Mazzolini R., Dopeso H., Mateo-Lozano S., Chang W., Rodrigues P., Bazzocco S., Alazzouzi H., Landolfi S., Hernandez-Losa J., Andretta E. (2012). Brush border Myosin Ia has tumor suppressor activity in the intestine. Proc. Natl. Acad. Sci. USA.

[31] Ouderkirk-Pecone J.L., Goreczny G.J., Chase S.E., Tatum A.H., Turner C.E., Krendel M. (2016). Myosin 1e promotes breast cancer malignancy by enhancing tumor cell proliferation and stimulating tumor cell de-differentiation. Oncotarget.

[32] Ohmura G., Tsujikawa T., Yaguchi T., Kawamura N., Mikami S., Sugiyama J., Nakamura K., Kobayashi A., Iwata T., Nakano H. (2015). Aberrant Myosin 1b Expression Promotes Cell Migration and Lymph Node Metastasis of HNSCC. Mol. Cancer Res..

